# Supplementary material for: Emerging Southeast Asian PfCRT mutations confer Plasmodium falciparum resistance to the first-line antimalarial piperaquine
Source: Nat Commun. 2018 Aug 17;9:3314. doi: 10.1038/s41467-018-05652-0 (PMC6095916; doi:10.1038/s41467-018-05652-0)
Supplement: Supplementary file 1 — Supplementary Information [file 41467_2018_5652_MOESM1_ESM.pdf]

Supplementary Information

Emerging Southeast Asian PfCRT mutations confer *Plasmodium falciparum* resistance to the first-line antimalarial piperaquine

Leila S. Ross, Satish K. Dhingra, Sachel Mok, Tomas Yeo, Kathryn J. Wicht, Krittikorn Kumpornsin, Shannon Takala-Harrison, Benoit Witkowski, Rick M. Fairhurst, Fred Ariey, Didier Menard, David A. Fidock

Supplementary Figure 1 | Chemical structures of antimalarial compounds used in this study.
Supplementary Figure 2 | Year of sampling of *P. falciparum* isolates of Asian origin, included in the *pfcrt* sequence analysis of the Pf3K release 3 genome data plus a more recent deposit of 87 genomes from Cambodia.
Supplementary Figure 3 | Zinc-Finger Nuclease (ZFN)-mediated editing of the *pfcrt* gene.
Supplementary Table 1 | Frequency distribution of PfCRT haplotypes from the Pf3K dataset across Asia.
Supplementary Table 2 | PfCRT haplotypes from the Pf3K dataset in Southeast Asia.
Supplementary Table 3 | Western Cambodian PfCRT haplotypes over time and K13 status.
Supplementary Table 4 | $PSA_{0\text{-}3h}$ Results.
Supplementary Table 5 | Piperaquine IC_{50} and IC_{90} values and associated statistics.
Supplementary Table 6 | [^{3}H]-Piperaquine cellular accumulation ratios.
Supplementary Table 7 | Chloroquine and monodesethyl-chloroquine IC_{50} values and associated statistics.
Supplementary Table 8 | Monodesethyl-amodiaquine and quinine IC_{50} values and associated statistics.
Supplementary Table 9 | Mefloquine and lumefantrine IC_{50} values and associated statistics.
Supplementary Table 10 | Dihydroartemisinin IC_{50} values and associated statistics.
Supplementary Table 11 | Verapamil reversibility of chloroquine and piperaquine IC_{50} values.
Supplementary Table 12 | Raw fitness data from co-culture experiments.
Supplementary Table 13 | *pfpm2* copy number and RNA expression analysis.
Supplementary Table 14 | List of oligonucleotides used in this study.

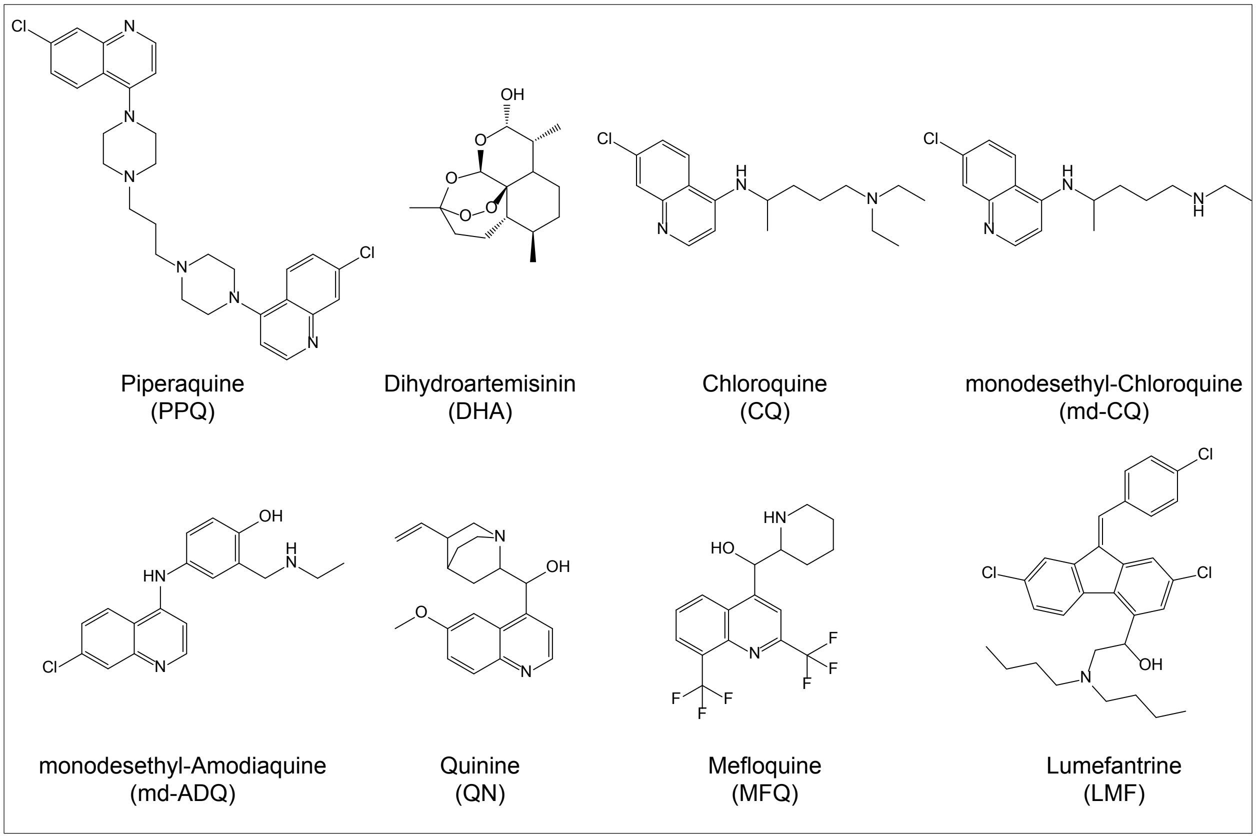

Supplementary Figure 1 | Chemical structures of compounds used in this study. These compounds represent current (PPQ, DHA, ADQ, MFQ, LMF) and prior (CQ, QN) first-line drugs used for the treatment of *P. falciparum* malaria. md-CQ and md-ADQ are the active metabolites of CQ and ADQ, respectively.

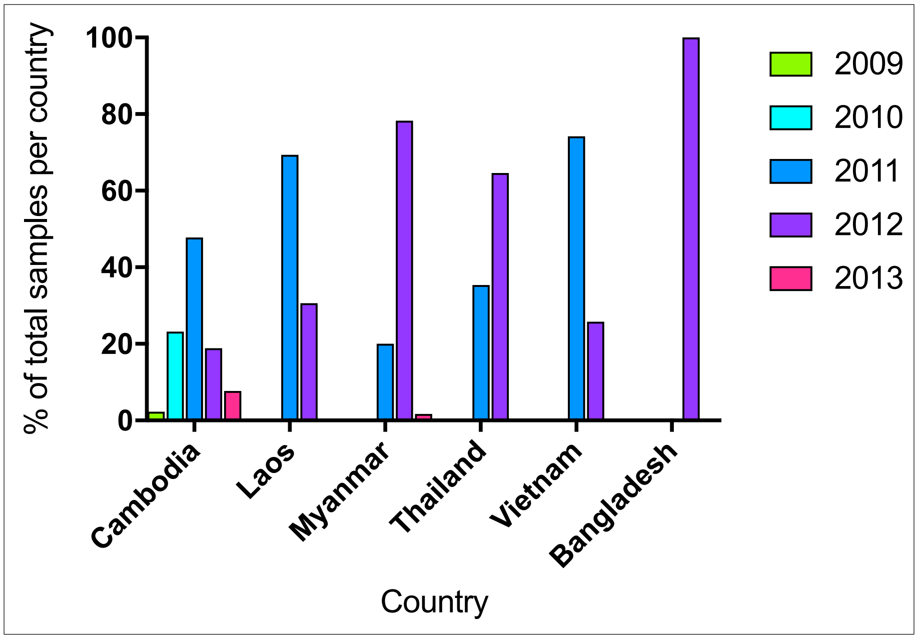

Supplementary Figure 2 | Year of sampling of *P. falciparum* isolates of Asian origin, included in the *pfcrt* sequence analysis of the Pf3K release 3 genome data plus a more recent deposit of 87 genomes from Cambodia. Most samples were collected in 2011–12, with additional samples from 2009–13 in Cambodia. The total number of genomes analyzed was 869. Sample numbers per country are listed in **Supplementary Table 1**.

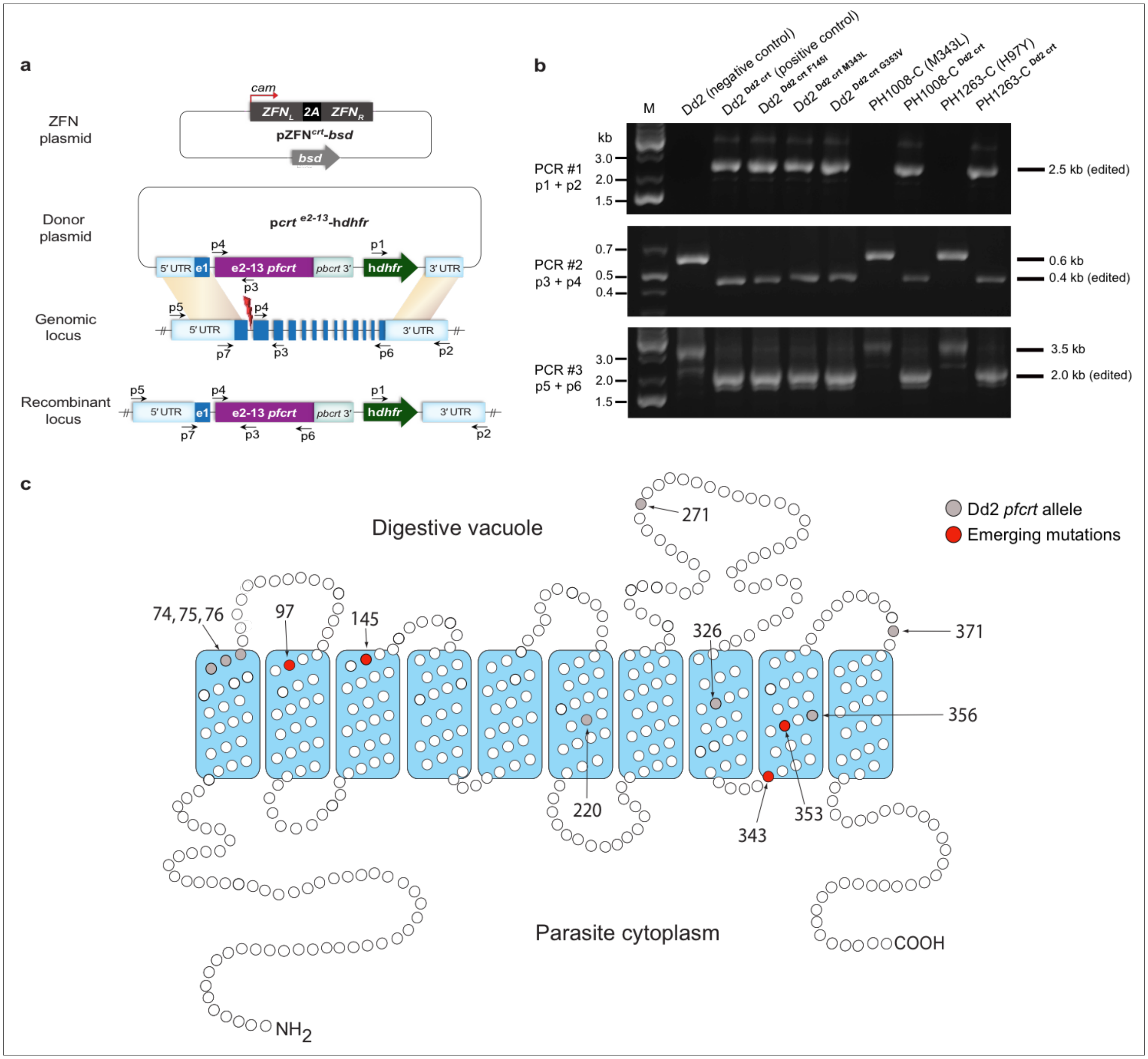

Supplementary Figure 3 | Zinc-Finger Nuclease (ZFN)-mediated editing of the *pfcrt* gene. (**a**) A two-plasmid transfection approach was used to edit the *pfcrt* gene with zinc-finger nucleases (ZFNs). (**b**) Three sets of PCRs were used to indicate gene editing, and the resulting modified locus was then verified by Sanger sequencing. (**c**) Schematic of the proposed PfCRT structure with ten transmembrane domains shows the relative locations of the mutations that make up the Dd2 *pfcrt* allele (gray) and the emerging Cambodian mutations that we showed by gene editing experiments are associated with PPQ resistance (red). Additional mutations detected in SE Asia, predominantly in Cambodia, are shown in **Supplementary Table 2**.

Supplementary Table 1 | Frequency distribution of PfCRT haplotypes from the Pf3K dataset across Asia.

**Isoform**	**# of variants**	**No. of isolate**	**Total %**	**Cambodia**	**%**	**Laos**	**%**	**Myanmar**	**%**	**Thailand**	**%**	**Vietnam**	**%**	**Bangladesh**	**%**
3D7 (wild-type)	0	**7**	**0.8%**	6	1.2%	-	-	-	-	-	-	1	1.2%	-	-
Dd2	8	**501**	**57.7%**	265	52.1%	12	17.6%	51	98.1%	122	96.1%	39	48.1%	12	37.5%
Dd2 + T93S (Novel)	9	**3**	**0.3%**	3	0.6%	-	-	-	-	-	-	-	-	-	-
Dd2 + H97L (Novel)	9	**9**	**1.0%**	8	1.6%	-	-	-	-	1	0.8%	-	-	-	-
**Dd2 + H97Y (Novel)**	9	**19**	**2.2%**	19	3.7%	-	-	-	-	-	-	-	-	-	-
**Dd2 + F145I (Novel)**	9	**1**	**0.1%**	1	0.2%	-	-	-	-	-	-	-	-	-	-
Dd2 + L196P (Novel)	9	**2**	**0.2%**	0	0.0%	-	-	-	-	2	1.6%	-	-	-	-
Dd2 + I218F (Novel)	9	**11**	**1.3%**	11	2.2%	-	-	-	-	-	-	-	-	-	-
Dd2 + T256I (Novel)	9	**4**	**0.5%**	4	0.8%	-	-	-	-	-	-	-	-	-	-
Dd2 + N295I (Novel)	9	**1**	**0.1%**	1	0.2%	-	-	-	-	-	-	-	-	-	-
**Dd2 + M343L (Novel)**	9	**4**	**0.5%**	4	0.8%	-	-	-	-	-	-	-	-	-	-
**Dd2 + G353V (Novel)**	9	**9**	**1.0%**	9	1.8%	-	-	-	-	-	-	-	-	-	-
Dd2 + A366T (Novel)	9	**3**	**0.3%**	3	0.6%	-	-	-	-	-	-	-	-	-	-
Dd2 + G367C (Novel)	9	**2**	**0.2%**	2	0.4%	-	-	-	-	-	-	-	-	-	-
Dd2 + V370C (Novel)	9	**1**	**0.1%**	1	0.2%	-	-	-	-	-	-	-	-	-	-
Dd2 - N75E (Novel)	7	**5**	**0.6%**	3	0.6%	-	-	-	-	-	-	2	2.5%	-	-
Dd2 - A220S (Novel)	7	**3**	**0.3%**	3	0.6%	-	-	-	-	-	-	-	-	-	-
Cam734	9	**123**	**14.2%**	71	13.9%	25	36.8%	-	-	-	-	27	33.3%	-	-
GB4	6	**111**	**12.8%**	75	14.7%	28	41.2%	-	-	-	-	8	9.9%	-	-
GB4 + I194T (Novel)	7	**11**	**1.3%**	8	1.6%	3	4.4%	-	-	-	-	-	-	-	-
GB4 - N75E (Novel)	5	**4**	**0.5%**	1	0.2%	-	-	-	-	-	-	3	3.7%	-	-
GB4 - A220S (Novel)	5	**2**	**0.2%**	2	0.4%	-	-	-	-	-	-	-	-	-	-
Cam783	7	**23**	**2.6%**	2	0.4%	-	-	-	-	-	-	1	1.2%	20	62.5%
Cam738	8	**3**	**0.3%**	3	0.6%	-	-	-	-	-	-	-	-	-	-
Cam738 + N75E (Novel)	8	**5**	**0.6%**	4	0.8%	-	-	-	-	1	0.8%	-	-	-	-
FCB	7	**2**	**0.2%**	0	0.0%	-	-	1	1.9%	1	0.8%	-	-	-	-
Total		869	100.0%	509		68		52		127		81		32	
Percentage of total				58.6%		7.8%		6.0%		14.6%		9.3%		3.7%	

Samples were collected from the Pf3K data version 3 (totaling 782 genomes that passed our analysis), supplemented with an additional 87 Cambodian genomes collected in 2012 and 2013 that were more recently deposited by the Pf3K consortium. This resulted in a total of 869 genomes, of which 509 were from Cambodia. A dash indicates that the allele was not observed in any genomes from that country.

Supplementary Table 2 | PfCRT haplotypes from the Pf3K dataset in Southeast Asia.

Isoform	74	75	76	93	97	144	145	148	194	196	218	220	224	228	256	271	295	321	326	333	343	353	356	366	367	370	371
3D7 (wild-type)	M	N	K	T	H	A	F	L	I	L	I	A	V	N	T	Q	N	L	N	T	M	G	I	A	G	V	R
Dd2	I	E	T	T	H	A	F	L	I	L	I	S	V	N	T	E	N	L	S	T	M	G	T	A	G	V	I
Dd2 + T93S (Novel)	I	E	T	S	H	A	F	L	I	L	I	S	V	N	T	E	N	L	S	T	M	G	T	A	G	V	I
Dd2 + H97L (Novel)	I	E	T	T	L	A	F	L	I	L	I	S	V	N	T	E	N	L	S	T	M	G	T	A	G	V	I
**Dd2 + H97Y (Novel)**	I	E	T	T	Y	A	F	L	I	L	I	S	V	N	T	E	N	L	S	T	M	G	T	A	G	V	I
**Dd2 + F145I (Novel)**	I	E	T	T	H	A	I	L	I	L	I	S	V	N	T	E	N	L	S	T	M	G	T	A	G	V	I
Dd2 + L196P (Novel)	I	E	T	T	H	A	F	L	I	P	I	S	V	N	T	E	N	L	S	T	M	G	T	A	G	V	I
Dd2 + I218F (Novel)	I	E	T	T	H	A	F	L	I	L	F	S	V	N	T	E	N	L	S	T	M	G	T	A	G	V	I
Dd2 + T256I (Novel)	I	E	T	T	H	A	F	L	I	L	I	S	V	N	I	E	N	L	S	T	M	G	T	A	G	V	I
Dd2 + N295I (Novel)	I	E	T	T	H	A	F	L	I	L	I	S	V	N	T	E	I	L	S	T	M	G	T	A	G	V	I
**Dd2 + M343L (Novel)**	I	E	T	T	H	A	F	L	I	L	I	S	V	N	T	E	N	L	S	T	L	G	T	A	G	V	I
**Dd2 + G353V (Novel)**	I	E	T	T	H	A	F	L	I	L	I	S	V	N	T	E	N	L	S	T	M	V	T	A	G	V	I
Dd2 + A366T (Novel)	I	E	T	T	H	A	F	L	I	L	I	S	V	N	T	E	N	L	S	T	M	G	T	T	G	V	I
Dd2 + G367C (Novel)	I	E	T	T	H	A	F	L	I	L	I	S	V	N	T	E	N	L	S	T	M	G	T	A	C	V	I
Dd2 + V370C (Novel)	I	E	T	T	H	A	F	L	I	L	I	S	V	N	T	E	N	L	S	T	M	G	T	A	G	C	I
Dd2 - N75E (Novel)	I	N	T	T	H	A	F	L	I	L	I	S	V	N	T	E	N	L	S	T	M	G	T	A	G	V	I
Dd2 - A220S (Novel)	I	E	T	T	H	A	F	L	I	L	I	A	V	N	T	E	N	L	S	T	M	G	T	A	G	V	I
Cam734	I	D	T	T	H	F	F	I	T	L	I	S	V	N	T	E	N	L	N	S	M	G	I	A	G	V	R
GB4	I	E	T	T	H	A	F	L	I	L	I	S	V	N	T	E	N	L	N	T	M	G	I	A	G	V	I
GB4 + I194T (Novel)	I	E	T	T	H	A	F	L	T	L	I	S	V	N	T	E	N	L	N	T	M	G	I	A	G	V	I
GB4 - N75E (Novel)	I	N	T	T	H	A	F	L	I	L	I	S	V	N	T	E	N	L	N	T	M	G	I	A	G	V	I
GB4 - A220S (Novel)	I	E	T	T	H	A	F	L	I	L	I	A	V	N	T	E	N	L	N	T	M	G	I	A	G	V	I
Cam783	I	E	T	T	H	A	F	L	I	L	I	S	V	N	T	E	N	L	N	T	M	G	T	A	G	V	I
Cam738	I	D	T	T	H	A	F	I	T	L	I	S	V	N	T	E	N	L	N	S	M	G	I	A	G	V	R
Cam738 + N75E (Novel)	I	E	T	T	H	A	F	I	T	L	I	S	V	N	T	E	N	L	N	S	M	G	I	A	G	V	R
FCB	I	E	T	T	H	A	F	L	I	L	I	S	V	N	T	E	N	L	S	T	M	G	I	A	G	V	I

Differences from the 3D7 wild-type *pfcrt* allele are highlighted in light grey for Dd2 mutations and dark grey for other mutations.

Supplementary Table 3 | Western Cambodian PfCRT haplotypes over time and K13 status.

	2010		2011		2012		2013		2016		K13 Haplotypes by Percentage				
**Isoform**	**#**	**%**	**#**	**%**	**#**	**%**	**#**	**%**	**#**	**%**	**Y493H**	**R539T**	**I543T**	**C580Y**	**WT**
3D7 (wild-type)	-	-	-	-	-	-	-	-	-	-	-	-	-	-	
Dd2	6	75	25	89	14	58	8	67	1	4.8	3.7%	22.2%	1.9%	63%	9.3%
Dd2 + T93S (Novel)	-	-	-	-	-	-	-	-	2	9.5	-	-	-	100%	-
Dd2 + H97L (Novel)	-	-	-	-	2	8.3	-	-	-	-	-	-	-	100%	-
**Dd2 + H97Y (Novel)**	-	-	1	3.6	-	-	-	-	3	14	-	-	-	100%	-
**Dd2 + F145I (Novel)**	-	-	-	-	-	-	-	-	1	4.8	-	-	-	100%	-
Dd2 + I218F (Novel)	-	-	-	-	-	-	-	-	2	9.5	-	-	-	100%	-
**Dd2 + M343L (Novel)**	-	-	-	-	5	21	4	33	-	-	-	-	-	100%	-
**Dd2 + G353V (Novel)**	-	-	-	-	2	8.3	-	-	12	57.1	-	-	-	100%	-
GB4	1	13	2	7.1	1	4.2	-	-	-	-	-	-	-	-	100%
Cam783	1	13	-	-	-	-	-	-	-	-	-	-	-	-	100%
Total	8		28		24		12		21		2	12	1	68	10

Data were generated from complete genome sequences of 93 Cambodian isolates collected by researchers at the Pasteur Institute in Cambodia from Pailin, Kampong Speu, Kampong Som, Kampong Thom, Oddar Meanchey, Battambang, Pursat and Sieam Reap in the western half of Cambodia. 19 of the samples from 2016 were from Pursat, and all harbored novel PfCRT mutations.

Supplementary Table 4 | $PSA_{0\text{-}3h}$ results.

Parasite line	% Survival	N	Notation	*p* value
3D7	0.3 ± 0.2	4	n.s.	>0.99
GC03	0.4 ± 0.2	4	n.s.	>0.99
Dd2	0.3 ± 0.2	4	n.s.	>0.99
Dd2$^{\text{Dd2 crt}}$	0.3 ± 0.3	4	N.A.	N.A.
Dd2$^{\text{Dd2 crt F145I}}$	23.4 ± 2.1	6	**	0.010[a]
Dd2$^{\text{Dd2 crt M343L}}$	12.4 ± 2.1	6	**	0.010[a]
Dd2$^{\text{Dd2 crt G353V}}$	16.7 ± 4.7	5	*	0.016[a]
PH1008-C (M343L)	14.4 ± 1.5	6	N.A.	N.A.
PH1008-C$^{\text{Dd2 crt}}$	0.1 ± 0.1	4	**	0.010[b]
PH1263-C (H97Y)	14.5 ± 4.3	4	N.A.	N.A.
PH1263-C$^{\text{Dd2 crt}}$	0.03 ± 0.02	4	**	0.010[c]

$PSA_{0\text{-}3h}$, piperaquine survival assays beginning with 0-3h post-invasion rings. Percent survival rates were calculated as means ± SEM from multiple independent experiments (N) performed in duplicate. [a]Statistical comparisons were made against the Dd2$^{\text{Dd2 crt}}$ clone. [b]Comparisons were against PH1008-C. [c]Comparisons were against PH1263-C. Statistics employed Mann-Whitney *U* tests. n.s., not significant. N.A., not applicable. 1 to 3 assays were also performed with 0.8 μM VP, which showed no change or net reduction in survival in either sensitive or resistant parasites.

Supplementary Table 5 | Piperaquine IC_{50} and IC_{90} values and associated statistics.

	Mean ± SEM		Statistical outputs	
Parasite line	PPQ IC_{50} (nM)	N	Notation	*p* value
3D7	13.7 ± 1.1	9	***	0.0002[a]
GC03	15.3 ± 2.2	5	*	0.012[a]
Dd2	22.4 ± 2.1	8	n.s.	0.37[a]
$Dd2^{Dd2\ crt}$	27.6 ± 3.1	9	N.A.	N.A.
$Dd2^{Dd2\ crt\ F145I}$	65.3 ± 3.1	4	**	0.003[a]
$Dd2^{Dd2\ crt\ M343L}$	37.6 ± 4.8	6	n.s.	0.11[a]
$Dd2^{Dd2\ crt\ G353V}$	31.8 ± 7.4	5	n.s.	0.70[a]
PH1008-C	39.6 ± 18.5	4	N.A.	N.A.
PH1008-$C^{Dd2\ crt}$	21.3 ± 3.0	5	n.s.	1.00[b]
PH1263-C	81.2 ± 26.0	4	N.A.	N.A.
PH1263-$C^{Dd2\ crt}$	18.0 ± 3.0	4	*	0.029[c]

[a]Statistical comparisons were made against the $Dd2^{Dd2\ crt}$ clone. [b]Comparisons were against PH1008-C. [c]Comparisons were against PH1263-C. N, number of independent repeats (assays conducted with technical duplicates). Statistics employed Mann-Whitney *U* tests. PPQ, piperaquine. n.s., not significant. N.A., not applicable.

	Mean ± SEM		Statistical outputs	
Parasite line	PPQ IC_{90} (nM)	N	Notation	*p* value
3D7	21.9 ± 2.2	9	****	<0.0001[a]
GC03	25.8 ± 2.9	5	*	0.012[a]
Dd2	45.5 ± 5.1	8	n.s.	0.48[a]
$Dd2^{Dd2\ crt}$	50.2 ± 4.2	9	N.A.	N.A.
$Dd2^{Dd2\ crt\ F145I}$	5769 ± 666	4	**	0.003[a]
$Dd2^{Dd2\ crt\ M343L}$	93.1 ± 9.7	6	***	0.0004[a]
$Dd2^{Dd2\ crt\ G353V}$	3741 ± 1981	4	**	0.003[a]
PH1008-C	> 50 μM	4	N.A.	N.A.
PH1008-$C^{Dd2\ crt}$	42.4 ± 6.2	5	****	<0.0001[b]
PH1263-C	250.8 ± 95.4	4	N.A.	N.A.
PH1263-$C^{Dd2\ crt}$	27.8 ± 3.0	4	*	0.029[c]

Please refer to footnotes in upper table.

Supplementary Table 6 | [^{3}H]-Piperaquine cellular accumulation ratios.

Parasite line	Mean ± SEM CAR	N	Statistical outputs Notation	*p* value
3D7	6323 ± 352	7	****	<0.0001[a]
GC03	4933 ± 229	4	***	0.0005[a]
Dd2	2835 ± 244	6	n.s.	0.73
Dd2$^{Dd2\ crt}$	3002 ± 275	7	N.A.	N.A.
Dd2$^{Dd2\ crt\ F145I}$	2878 ± 327	3	n.s.	0.72[a]
Dd2$^{Dd2\ crt\ M343L}$	2514 ± 231	6	n.s.	0.43[a]
Dd2$^{Dd2\ crt\ G353V}$	2642 ± 402	3	n.s.	0.21[a]
PH1008-C	1889 ± 163	4	N.A.	N.A.
PH1008-C$^{Dd2\ crt}$	3358 ± 121	4	***	0.0003[b]
PH1263-C	1690 ± 185	3	N.A.	N.A.
PH1263-C$^{Dd2\ crt}$	3029 ± 139	4	***	0.0007[c]

[a]Statistical comparisons were made against the Dd2$^{Dd2\ crt}$ clone. [b]Comparisons were against PH1008-C. [c]Comparisons were against PH1263-C. N, number of independent repeats (assays conducted with technical duplicates). Statistics employed Mann-Whitney *U* tests. CAR, cellular accumulation ratio. PPQ, piperaquine. n.s., not significant. N.A., not applicable.

Supplementary Table 7 | Chloroquine and monodesethyl-chloroquine IC_{50} values and associated statistics.

	Mean ± SEM		Statistical outputs	
Parasite line	CQ IC_{50} (nM)	N	Notation	*p* value
3D7	9.0 ± 0.8	8	****	<0.0001[a]
GC03	9.6 ± 2.1	5	***	0.005[a]
Dd2	156.5 ± 9.3	10	*	0.02[a]
$Dd2^{Dd2\ crt}$	129.5 ± 9.0	11	N.A.	N.A.
$Dd2^{Dd2\ crt\ F145I}$	19.3 ± 1.3	6	***	0.0002[a]
$Dd2^{Dd2\ crt\ M343L}$	41.8 ± 1.9	5	***	0.0005[a]
$Dd2^{Dd2\ crt\ G353V}$	54.6 ± 4.9	4	**	0.002[a]
PH1008-C	160.5 ± 8.0	10	N.A.	N.A.
PH1008-$C^{Dd2\ crt}$	112.7 ± 15.8	10	*	0.043[b]
PH1263-C	258.2 ± 38.9	6	N.A.	N.A.
PH1263-$C^{Dd2\ crt}$	170.4 ± 25.5	6	n.s.	0.065[c]

[a]Statistical comparisons were made against the $Dd2^{Dd2\ crt}$ clone. [b]Comparisons were against PH1008-C. [c]Comparisons were against PH1263-C. N, number of independent repeats (assays conducted with technical duplicates). Statistics employed Mann-Whitney *U* tests. CQ, chloroquine. n.s., not significant. N.A., not applicable.

	Mean ± SEM		Statistical outputs	
Parasite line	md-CQ IC_{50} (nM)	N	Notation	*p* value
3D7	33.1 ± 5.7	10	****	<0.0001[a]
GC03	52.8 ± 9.5	6	****	<0.0001[a]
Dd2	1663 ± 141	10	n.s.	0.98[a]
$Dd2^{Dd2\ crt}$	1641 ± 108	15	N.A.	N.A.
$Dd2^{Dd2\ crt\ F145I}$	191 ± 21.3	4	***	0.0005[a]
$Dd2^{Dd2\ crt\ M343L}$	457 ± 25.5	6	****	<0.0001[a]
$Dd2^{Dd2\ crt\ G353V}$	475 ± 57.7	6	****	<0.0001[a]
PH1008-C	1418 ± 259	4	N.A.	N.A.
PH1008-$C^{Dd2\ crt}$	1527 ± 270	5	n.s.	0.90[b]
PH1263-C	3313 ± 435	4	N.A.	N.A.
PH1263-$C^{Dd2\ crt}$	1345 ± 229	5	*	0.016[c]

Please refer to footnotes in upper table. md-CQ, monodesethyl-chloroquine.

Supplementary Table 8 | Monodesethyl-amodiaquine and quinine IC_{50} values and associated statistics.

Parasite line	Mean ± SEM md-ADQ IC_{50} (nM)	N	Statistical outputs Notation	*p* value
3D7	15.9 ± 1.9	10	****	<0.0001[a]
GC03	14 ± 1.2	6	****	<0.0001[a]
Dd2	45.6 ± 3.8	11	n.s.	0.12[a]
Dd2$^{Dd2\ crt}$	40.6 ± 2.4	15	N.A.	N.A.
Dd2$^{Dd2\ crt\ F145I}$	16.8 ± 1.5	5	***	0.0001[a]
Dd2$^{Dd2\ crt\ M343L}$	30.1 ± 2.1	6	*	0.014[a]
Dd2$^{Dd2\ crt\ G353V}$	23.0 ± 1.3	6	****	<0.0001[a]
PH1008-C	33.9 ± 3.5	6	N.A.	N.A.
PH1008-C$^{Dd2\ crt}$	42.7 ± 5.0	6	n.s.	0.31[b]
PH1263-C	63.9 ± 5.1	5	N.A.	N.A.
PH1263-C$^{Dd2\ crt}$	41.0 ± 2.9	6	**	0.009[c]

[a]Statistical comparisons were made against the Dd2$^{Dd2\ crt}$ clone. [b]Comparisons were against PH1008-C. [c]Comparisons were against PH1263-C. N, number of independent repeats (assays conducted with technical duplicates). Statistics employed Mann-Whitney tests. md-ADQ, monodesethyl-amodiaquine. n.s., not significant. N.A., not applicable.

Parasite line	Mean ± SEM QN IC_{50} (nM)	N	Statistical outputs Notation	*p* value
3D7	26.1 ± 2.2	14	****	<0.0001[a]
GC03	72.7 ± 12.7	7	***	0.0002[a]
Dd2	163.2 ± 22.9	14	n.s.	0.59[a]
Dd2$^{Dd2\ crt}$	174.2 ± 13.9	15	N.A.	N.A.
Dd2$^{Dd2\ crt\ F145I}$	57.4 ± 8.4	5	***	0.0001[a]
Dd2$^{Dd2\ crt\ M343L}$	93.9 ± 9.6	9	***	0.001[a]
Dd2$^{Dd2\ crt\ G353V}$	77.6 ± 15.1	6	**	0.002[a]
PH1008-C	61.8 ± 15.7	5	N.A.	N.A.
PH1008-C$^{Dd2\ crt}$	145.6 ± 28.1	6	*	0.035[b]
PH1263-C	44.2 ± 7.6	6	N.A.	N.A.
PH1263-C$^{Dd2\ crt}$	142.1 ± 22.5	6	**	0.002[c]

Please refer to footnotes in upper table. QN, quinine.

Supplementary Table 9 | Mefloquine and lumefantrine IC_{50} values and associated statistics.

	Mean ± SEM		Statistical outputs	
Parasite line	MFQ IC_{50} (nM)	N	Notation	*p* value
3D7	9.7 ± 1.3	15	n.s.	0.43[a]
GC03	9.2 ± 1.0	8	n.s.	0.42[a]
Dd2	12.9 ± 1.3	12	n.s.	0.20[a]
$Dd2^{Dd2\ crt}$	10.1 ± 0.5	15	N.A.	N.A.
$Dd2^{Dd2\ crt\ F145I}$	7.4 ± 1.4	6	n.s.	0.20[a]
$Dd2^{Dd2\ crt\ M343L}$	7.3 ± 0.4	6	**	0.002[a]
$Dd2^{Dd2\ crt\ G353V}$	8.9 ± 0.5	6	n.s.	0.21[a]
PH1008-C	8.6 ± 1.4	5	N.A.	N.A.
PH1008-$C^{Dd2\ crt}$	13.9 ± 1.9	6	n.s.	0.13[b]
PH1263-C	3.5 ± 1	6	N.A.	N.A.
PH1263-$C^{Dd2\ crt}$	12.5 ± 2.1	6	**	0.004[c]

[a]Statistical comparisons were made against the $Dd2^{Dd2\ crt}$ clone. [b]Comparisons were against PH1008-C. [c]Comparisons were against PH1263-C. N, number of independent repeats (assays conducted with technical duplicates).Statistics employed Mann-Whitney *U* tests. MFQ, mefloquine. n.s., not significant. N.A., not applicable.

	Mean ± SEM		Statistical outputs	
Parasite line	LMF IC_{50} (nM)	N	Notation	*p* value
3D7	2.9 ± 0.6	9	n.s.	0.21[a]
GC03	3.9 ± 0.5	4	*	0.011[a]
Dd2	1.9 ± 0.3	8	n.s.	0.91[a]
$Dd2^{Dd2\ crt}$	1.8 ± 0.3	9	N.A.	N.A.
$Dd2^{Dd2\ crt\ F145I}$	1.6 ± 0.1	5	n.s.	1.00[a]
$Dd2^{Dd2\ crt\ M343L}$	3.7 ± 0.2	4	*	0.011[a]
$Dd2^{Dd2\ crt\ G353V}$	1.3 ± 0.2	7	n.s.	0.36[a]
PH1008-C	1.2 ± 0.2	4	N.A.	N.A.
PH1008-$C^{Dd2\ crt}$	4.4 ± 1.2	5	*	0.016[b]
PH1263-C	3.6 ± 0.7	4	N.A.	N.A.
PH1263-$C^{Dd2\ crt}$	8.0 ± 0.5	4	*	0.029[c]

Please refer to footnotes in upper table. LMF, lumefantrine.

Supplementary Table 10 | Dihydroartemisinin IC_{50} values and associated statistics.

	Mean ± SEM		Statistical outputs	
Parasite line	DHA IC_{50} (nM)	N	Notation	*p* value
3D7	0.8 ± 0.1	11	**	0.004[a]
GC03	0.5 ± 0.2	5	**	0.007[a]
Dd2	1.0 ± 0.1	10	n.s.	0.25[a]
Dd2$^{Dd2\ crt}$	1.2 ± 0.1	9	N.A.	N.A.
Dd2$^{Dd2\ crt\ F145I}$	0.7 ± 0.1	6	***	0.0008[a]
Dd2$^{Dd2\ crt\ M343L}$	0.6 ± 0.1	5	***	0.001[a]
Dd2$^{Dd2\ crt\ G353V}$	1.3 ± 0.3	4	n.s.	1.00[a]
PH1008-C	1.5 ± 0.2	4	N.A.	N.A.
PH1008-C$^{Dd2\ crt}$	0.6 ± 0.1	4	*	0.029[b]
PH1263-C	2.4 ± 0.1	4	N.A.	N.A.
PH1263-C$^{Dd2\ crt}$	1.9 ± 0.3	4	n.s.	0.29[c]

[a]Statistical comparisons were made against the Dd2$^{Dd2\ crt}$ clone. [b]Comparisons were against PH1008-C. [c]Comparisons were against PH1263-C. N, number of independent repeats (assays conducted with technical duplicates). Statistics employed Mann-Whitney *U* tests. DHA, dihydroartemisinin. n.s., not significant. N.A., not applicable.

Supplementary Table 11 | Verapamil reversibility of chloroquine and piperaquine IC_{50} values.

Parasite line	CQ reversibility	N	Statistics compared to 3D7		Statistics compared to parent		% VP inhibition[d]
			Notation	*p* value	Notation	*p* value	
3D7	0.5% ± 0.5%	4	N.A.	--	N.A.	--	8.9% ± 3.2%
GC03	0.5% ± 0.5%	4	n.s.	>0.99	N.A.	--	6.6% ± 3.5%
Dd2	71.8% ± 3.0%	4	*	0.029	n.s.	0.71[a]	7.8% ± 4.9%
Dd2[Dd2 crt]	72.8% ± 1.3%	4	*	0.029	N.A.	--	8.7% ± 1.4%
Dd2[Dd2 crt F145I]	25.8% ± 10.8%	6	n.s.	0.11	**	0.010[a]	45.2% ± 5.6%
Dd2[Dd2 crt M343L]	43.3% ± 10.9%	4	*	0.029	*	0.029[a]	33.7% ± 10.4%
Dd2[Dd2 crt G353V]	53.3% ±3.4%	6	*	0.029	**	0.010[a]	35.5% ± 7.6%
PH1008-C	62.8% ± 1.7%	4	*	0.029	N.A.	--	18.0% ± 4.1%
PH1008-C[Dd2 crt]	65.3% ± 1.4%	4	*	0.029	n.s.	0.63[b]	13.2% ± 4.4%
PH1263-C	59.8% ± 4.5%	6	**	0.010	N.A.	--	22.4% ± 3.4%
PH1263-C[Dd2 crt]	63.8% ± 2.6%	4	*	0.029	n.s.	0.35[c]	14.2% ± 1.7%

Reversibility is defined as the percent reduction in mean IC_{50} for CQ in the presence of 0.8 μM VP compared to the mean IC_{50} for CQ alone. The calculation is defined as ((mean IC_{50} CQ alone - mean IC_{50} CQ + VP) / mean IC_{50} CQ alone), expressed as a percentage. [a]Statistical comparisons were made against the Dd2[Dd2 crt] clone. [b]Comparisons were against PH1008-C. [c]Comparisons were against PH1263-C. [d] %VP inhibition measures the percent inhibition of parasite growth in the presence of 0.8 μM VP compared to no-drug controls no CQ). N, number of independent repeats (assays conducted with technical duplicates). Statistics employed Mann-Whitney *U* tests. n.s., not significant. N.A., not applicable.

Parasite line	PPQ reversibility	N	Statistics compared to 3D7		Statistics compared to parent	
			Notation	*p* value	Notation	*p* value
3D7	7.8% ± 4.5%	4	N.A.	--	N.A.	--
GC03	17.0% ± 5.7%	4	ns	0.49	N.A.	--
Dd2	2.0% ± 4.5%	4	ns	0.49	ns	0.89[a]
Dd2[Dd2 crt]	5.0% ± 9.2%	4	ns	0.94	N.A.	--
Dd2[Dd2 crt F145I]	16.6% ± 17.4%	5	ns	0.73	ns	0.56[a]
Dd2[Dd2 crt M343L]	32.5% ± 5.0%	4	*	0.029	ns	0.057[a]
Dd2[Dd2 crt G353V]	43.4% ± 15.2%	3	ns	0.11	ns	0.23[a]
PH1008-C	N.D.	3	N.A.	--	N.A.	--
PH1008-C[Dd2 crt]	7.0% ± 8.0%	4	ns	0.97	N.A.	--
PH1263-C	N.D.	4	N.A.	--	N.A.	--
PH1263-C[Dd2 crt]	0.0% ± 6.2%	4	ns	0.49	N.A.	--

Please refer to footnotes in upper table. N.D., not determined (the flat dose-response curves around the IC_{50} region precluded precise calculations of VP reversiblity).

Supplementary Table 12 | Raw fitness data from co-culture experiments.

Day	GC03						Dd2					
0	48.05	42.35	49.37	50.28	52.87	47.59	40.91	37.84	44.55	44.23	47.34	46.39
2	50	49.46	48.39	67.95	64.95	64.66	43.62	42.94	43.9	51.81	58.92	50.4
5	80.43	81.64	80.67	53.1	48.35	50.16	61.06	59.29	60.96	45.45	43.61	39.15
7	77.64	75.39	74.31	39.82	35.8	39.63	35.53	36.47	38.69	27.78	27.6	28.13
9	60.8	58.89	61.22	32.74	35	30.86	17.99	17.84	19.63	14.51	17.78	14.29
12	44.23	46.65	45.88	33.01	30.17	31.33	16.22	14.46	14.44	13	11.01	10.44
14	47.34	45.7	48.72	31.93	29.81	28.66	10.03	9.61	6.98	10.68	9.41	9.21
16	47.84	51.06	54.59	16.73	21.79	16.69	6.02	7.32	6.03	5.69	3.83	4.88
18	40.49	46.88	44.64	19.59	20.36	19.41	5.28	5.69	6.08	8.32	8.84	8.09
20	46.21	49.59	45.87	15.2	18.22	14.77	5.92	5.21	6.12	6.5	5.15	6.92

Day	$Dd2^{Dd2\ crt}$						$Dd2^{Dd2\ crt\ F145I}$					
0	50	44.58	45.33	49.6	42.96	46.92	51.95	50	53.95	50.83	45.45	52.99
2	56.67	53.92	50.63	67.79	69.61	63.84	54.23	52.43	55.84	36.36	34.03	34.3
5	74.6	74.76	72.64	62.57	67.64	52.2	87.1	88.06	87.78	52.32	51.26	48.68
7	71.5	72.59	62.1	58.17	59.43	38.1	95.77	96.29	94.47	69.81	67.73	65.15
9	47.9	54.43	40.4	30.71	28.73	19.42	91.45	92.25	91.29	65.5	61.49	60.16
12	35.63	39.89	22.78	28.5	24.92	15.69	79.92	78.83	82.06	54.88	55.17	54.39
14	34.97	33.82	15.41	22.55	20.37	11.25	85.71	87.71	92.03	62	57.24	56.72
16	31.34	23.89	9.43	13.72	10.46	7.09	91.53	92.34	92.12	66.67	64.41	71.11
18	25.43	16.03	5.56	12.43	10.57	7.81	93.55	95.2	94.19	79.82	81.98	80.02
20	20.03	18.64	7.16	9.46	7.05	5.36	94.48	95.47	94.94	89.67	89.16	91.2

Day	$Dd2^{Dd2\ crt\ M343L}$						$Dd2^{Dd2\ crt\ G353V}$					
0	38.33	45.53	42.86	44.55	48.13	47.51	46.67	44.35	55.21	49.5	48.73	47.92
2	46.94	45.55	45.71	60.75	61.15	55.88	56.36	55.43	57.95	66.59	70.69	68.82
5	75.72	78.45	77.53	69.93	51.35	52.65	83.75	83.58	80	68.83	69.23	70.69
7	68.09	73.2	52.52	23.89	24.68	33.11	70.59	74.07	71.87	68.67	70.78	69.62
9	14.85	10.3	16.36	13.86	23.68	21.88	62.07	70.18	69.76	54.23	54.85	52.98
12	14.29	8.45	13.45	13.37	14.03	21.9	75	74.26	70.37	64.12	62.92	60.58
14	6.99	4.05	7.73	7.52	12.76	17.11	61.6	71.31	70.39	67.28	66.15	66.24
16	3.64	2.71	7.14	4.56	9.14	11.63	62.8	68.13	71.65	64.85	67.16	66.41
18	2.46	1.15	4.6	6.38	11.99	14.04	60.94	67.33	65	67.13	73.17	72.21
20	2.17	1.45	3.97	3.39	8.14	12.42	60.33	70.86	73.27	65.78	68.87	60.77

Day	PH1008-C (M343L)						$PH1008\text{-}C^{Dd2\ crt}$					
0	42.35	49.44	42.68	61.45	61.9	61.45	42.62	50	38.35	48.72	47.18	44.62
2	63.68	66.74	67.39	81.27	79.77	84.35	53.18	53.83	53.24	69.46	65.54	62.42
5	96.69	95.24	96.72	89.84	89.17	88.2	92.28	91.95	92.75	54.75	55.54	52.65
7	95.9	95.78	95.36	92.31	90.77	91.24	66.38	70.27	70.5	52.62	48.67	42.52
9	97.34	96.03	96.18	91.38	91.99	91.78	53.57	55.35	56.06	58.35	54.55	41.85
12	99.02	98.26	96.36	95.17	94.78	96.84	48.5	49.24	40.15	54.19	51.59	31.37
14	96.61	96.37	95.65	95.68	96.83	96.58	44.95	42.94	30.59	54.65	51.14	26.96
16	89.47	95.28	95.58	89.84	92.03	94.11	46.78	44.89	33.38	51.49	49.02	21.46
18	81.64	84.64	93.57	94.62	97.62	96.08	56.67	54.42	32.07	52.09	46.27	26.11
20	94.75	94.22	97.45	91.81	93	93.37	45.09	42.29	33.33	57.63	50.17	32.13

Day	PH1263-C (H97Y)						$PH1263\text{-}C^{Dd2\ crt}$					
0	54.1	44.44	48.98	52.63	46.67	48.94	52.14	46.09	48.18	42.62	41.67	46.89
2	65.67	63.52	61.72	70.42	77.53	77.97	56.66	56.99	56.57	68.65	65.67	66.02
5	95.48	98.2	96.69	84.85	88.45	90.17	96.15	94.71	92.38	57.67	59.88	57.79
7	94.98	97.15	96.17	88.64	95.65	93.33	82.67	68.62	73.38	57.13	61.3	56.65
9	95.76	96.97	97.92	91.15	91.8	91.33	53.15	56.57	60.57	52.88	51.03	54.29
12	97.87	97.47	98.66	93.35	96.17	95.64	49.06	52.08	56.41	55.04	59.48	59.61
14	94.34	95.06	96.15	95.32	95.58	95.9	41.06	50.88	64.57	53.09	61.9	63.22
16	95.28	94.92	95.31	89.25	90.68	92.18	41.67	53.18	66.88	52.54	57.72	55.4
18	94	93.57	90.11	95.79	96.88	96.89	47.44	48.48	52.94	56.05	63.72	62.29
20	96.45	96.48	96.87	89.76	89.11	93.74	52.19	61.9	52.66	63.36	65.76	65.02

Two independent experiments were performed in triplicate. The values listed are the % eGFP+ cells.

Supplementary Table 13 | *pfpm2* copy number and RNA expression analysis.

Ratios *pfpm2* : Pf β-tubulin	qPCR (normalized by *pfB-tubulin*) Mean	SEM	Calculated *pfpm2* copy number	N	RT-qPCR (normalized by *serine-tRNA ligase*) Mean RNA expression over 3D7 control	SEM	*pfpm2* expression in edited line over parental line	N
1:1	0.93	0.04	1	3	na	na	na	na
2:1	2.22	0.05	2	3	na	na	na	na
3:1	2.91	0.04	3	3	na	na	na	na
4:1	3.97	0.10	4	3	na	na	na	na
5:1	4.19	0.08	5	3	na	na	na	na
3D7	1.00	0.00	1	3	1.00	0.00	na	3
GC03	1.02	0.04	1	3	1.33	0.08	na	3
Dd2	1.10	0.06	1	6	1.50	0.06	na	3
$Dd2^{Dd2\ crt}$	1.16	0.08	1	6	1.25	0.18	1.00	6
$Dd2^{Dd2\ crt\ F145I}$	1.07	0.02	1	3	1.31	0.10	1.05	3
$Dd2^{Dd2\ crt\ M343L}$	1.04	0.04	1	3	1.01	0.06	0.81	3
$Dd2^{Dd2\ crt\ G353V}$	1.14	0.02	1	3	0.31#	0.03	0.25#	3
PH1008-C	3.02	0.06	3	6	2.59	0.20	na	3
$PH1008\text{-}C^{Dd2\ crt}$	1.23	0.01	1	3	0.87	0.15	0.34	3
PH1263-C	1.80	0.03	2	3	1.41	0.04	na	3
$PH1263\text{-}C^{Dd2\ crt}$	0.79	0.02	1	3	0.51	0.03	0.36	3

pfpm2 copy number and expression levels at the mid trophozoite stage (24 hours post-invasion) were determined using multiplexed Taqman assays (see Methods). na: not applicable. #: sample was collected earlier in early trophs and late rings when *pfpm2* expression is lower, hence change was not significant.

Supplementary Table 14 | List of oligonucleotides used in this study.

Name	Nucleotide Sequence (5'–3')	Description	Lab Name	Purpose
p1	gctcctaggATGCATGGTTCGCTAAACTGC	*hdhfr* AvrII Forward	p4725	Integration PCR #1. 2.5 kb yes/no integration at 3' end
p2	TTGACCCTTATATATTCCACCCA	*pfcrt* 3' UTR (+1285-1308)	p5688	
p3	GACCTTAACAGATGGCTCAC	*pfcrt* exon 2 EcoRI Forward	p3264	Integration PCR #2. 0.4 kb (0.6 kb if unedited due to additional intron 2). Sequences exons 2-3.
p4	cttatcgatAAGCAGAAGAACATATTAATAG GAATACTTAATTG	*pfcrt* exon 3 ClaI Reverse	p3265	
p5	cttgggCCCAAGTTGTACTGCTTCTAAGC	*pfcrt* 5' UTR (-494-517) ApaI Forward	p3404	Integration PCR #3. 2 kb. Sequence entire edited locus.
p6	aaccatggatTTATTGTGTAATAATTGAATC GACG	*pfcrt* exon 13 Reverse	p1640	
p7	CCGTTAATAATAAATACACGCAG	*pfcrt* 5'UTR sense	p4233	Sequencing primer for *pfcrt* (along with p3, p6).
p9	GTTTTGTAACATCCGAAACTtACAACTTT ATTTGTATGATTATGTTC	PfCRT H97Y sense SDM. CAC to TAC.	p5345	PfCRT H97Y SDM
p10	GAACATAATCATACAAATAAAGTTGTaA GTTTCGGATGTTACAAAAC	PfCRT H97Y antisense SDM. CAC to TAC.	p5346	
p11	CCTGTTCAGTCATTTTGGCCaTCATAGG TCTTACAAGAACTAC	PfCRT F145I sense SDM. TTC to ATC.	p6016	PfCRT F145I SDM
p12	GTAGTTCTTGTAAGACCTATGAtGGCCA AAATGACTGAACAGG	PfCRT F145I antisense SDM. TTC to ATC.	p6017	
p13	CGACAAATTTTCTACCtTGACATATACTA TTGTTAGTTG	PfCRT M343L sense SDM. ATG to TTG.	p5343	PfCRT M343L SDM
p14	CAACTAACAATAGTATATGTCAaGGTAG AAAATTTGTCG	PfCRT M343L antisense SDM. ATG to TTG.	p5344	
p15	CTATTGTTAGTTGTATACAAGtTCCAGCA ACAGCAATTG	PfCRT G353V sense SDM. GGT to GTT.	p5330	PfCRT G353V SDM
p16	CAATTGCTGTTGCTGGAaCTTGTATACA ACTAACAATAG	PfCRT G353V antisense SDM. GGT to GTT.	p5331	
p17	TGATGTGCGCAAGTGATCC	Pf β-tubulin sense qPCR primer	p5856	Pf β-tubulin qPCR
p18	TCCTTTGTGGACATTCTTCCTC	Pf β-tubulin antisense qPCR primer	p5957	
p19	HEX-TAGCACATGCCGTTAAATATCTTCCATG TCT-BHQ	Pf β-tubulin HEX and Black Hole Quencher dual-labeled probe	p5851	
p20	GGATTCGAACCAACTTATACTGC	Pf Plasmepsin 2 sense qPCR primer	p6758	Pf Plasmepsin 2 qPCR
p21	AATTGGATCTACTGAACCTATTGATAA	Pf Plasmepsin 2 antisense qPCR primer	p6759	
p22	FAM-CAACATTTGATGGTATCCTTGGTTTAGG ATGGA-MGB	Pf Plasmepsin 2 FAM and MGB dual-labeled probe	p6760	
p23	TGGAACAATGGTAGCTGCAC	Pf serine tRNA ligase sense qPCR primer	p6755	Pf serine tRNA ligase qPCR
p24	GGCGCAATTTTTCAGGAACT	Pf serine tRNA ligase antisense qPCR primer	p6756	
p25	HEX-TGTCTTCTTGAAAATTATCAAAACGGCG AAGG-BHG	Pf serine tRNA ligase HEX and Black Hole Quencher dual-labeled probe	p6757	

Lower-case nucleotides are not present in the template (restriction sites, modifying primer annealing temperature, or site-directed mutagenesis (SDM)).
